# Discovery of Endothelial–Monocyte Crosstalk in Ischemic‐Reperfusion Injury Following Liver Transplantation Based on Integration of Single‐Cell RNA and Transcriptome RNA Sequencing

**DOI:** 10.1111/jcmm.70336

**Published:** 2025-02-24

**Authors:** Chao Sun, Li Li, Dan Li, Zhengxin Wang

**Affiliations:** ^1^ Liver Transplantation Center, Department of General Surgery, Huashan Hospital Fudan University Shanghai China; ^2^ Institute of Organ Transplantation Fudan University Shanghai China; ^3^ Shanghai Institute of Immunology Shanghai Jiao Tong University School of Medicine Shanghai China

**Keywords:** cellular crosstalk, hepatic ischemia/reperfusion injury, liver sinusoidal endothelial cells, monocytes, single‐cell RNA sequencing

## Abstract

Hepatic ischemia/reperfusion injury (IRI) commonly complicates liver transplantation (LT). However, the precise mechanisms underlying hepatic IRI remain incompletely understood. We acquired single‐cell RNA sequencing (scRNA‐seq) and transcriptome RNA sequencing data of LT patients from the GEO database. Employing scRNA‐seq, we delved into the interplay between non‐immune and immune cells in hepatic IRI, pinpointing genes exhibiting altered expression patterns. Integrating insights gleaned from scRNA‐seq and transcriptome RNA sequencing datasets, we deepened our comprehension of cellular interactions and underlying mechanisms in hepatic IRI. Additionally, we conducted preliminary validation of identified gene expression alterations using immunofluorescence techniques. Using scRNA‐seq, we detected significant changes in the populations of liver sinusoidal endothelial cells (LSECs) and monocytes after hepatic ischemia–reperfusion injury (IRI). By integrating scRNA‐seq with bulk transcriptome RNA sequencing data, we identified key genes with dysregulated expression in LSECs (ICAM1, SOCS3, NFKBIZ, JUND, TNFRSF12A and HSPA6) and monocytes (SOCS3, JUND, FPR2 and NR4A2). Our analysis of cell communication indicated that the ANXA1‐FPR2 axis might be a pivotal signature in mediating interactions between LSECs and monocytes. We then established a mouse model for IRI, and further analyses using flow cytometry and immunofluorescence showed a significant increase in monocyte proportion post‐IR (*p* < 0.01). Consistently, Western Blot also revealed significant upregulation of ANXA1 and FPR2 (*p* < 0.01). Our study elucidated the cellular interactions and signalling pathways following IRI. The interplay between LSECs and monocytes likely triggers a cascade of events, promoting monocyte infiltration and amplifying inflammatory responses, thus worsening the deleterious effects of IRI.

**Abbreviations**
A1CF: APOBEC1 Complementation Factor
ACSL1: Acyl‐CoA Synthetase Long Chain Family Member 1
ANXA1: Annexin A1
AREG: Amphiregulin
ATF3: Activating Transcription Factor 3
ATF5: Activating Transcription Factor 5
BAG3: BCL2 Associated Athanogene 3
BCL2A1: BCL2 Related Protein A1
BHLHE40: Basic Helix–Loop–Helix Family Member E40
BMP2: Bone Morphogenetic Protein 2
BTG3: BTG Anti‐Proliferation Factor 3
C5AR1: Complement Component 5a Receptor 1
CARO1A: Calcium Regulated Oncogene 1A
CCL3: C‐C Motif Chemokine Ligand 3
CCL5: C‐C Motif Chemokine Ligand 5
CCNL1: Cyclin L1
CCR2: C‐C Chemokine Receptor Type 2
CD22: Cluster of Differentiation 22
CD4: Cluster of Differentiation 4
CD63: Cluster of Differentiation 63
CD74: Cluster of Differentiation 74
CDKN1A: Cyclin‐Dependent Kinase Inhibitor 1A
CXCL1: C‐X‐C Motif Chemokine Ligand 1
CXCR4: C‐X‐C Motif Chemokine Receptor 4
CYTOR: Cytosolic Tyrosine Aminotransferase 1
DEFA3: Defensin Alpha 3
DEGs: Differentially Expressed Genes
DEPP1: DEPP (DNA Damage Inducible Transcript 4) Homologue 1
DNAJB1: DnaJ Heat Shock Protein Family (Hsp40) Member B1
DNAJB4: DnaJ Heat Shock Protein Family (Hsp40) Member B4
DUSP5: Dual Specificity Phosphatase 5
EGFR: Epidermal Growth Factor Receptor
ELF3: E74 Like ETS Transcription Factor 3
ELMSAN1: ELM2 and SANT Domain Containing 1
EP: End of Preservation
ESRP1: Epithelial Splicing Regulatory Protein 1
ET1: Endothelin 1
ETV3: ETS Variant Transcription Factor 3
ETV6: ETS Variant Transcription Factor 6
EZR: Ezrin
FAM49A: Family with Sequence Similarity 49 Member A
FOS: Fos Proto‐Oncogene, AP‐1 Transcription Factor Subunit
FOS: Fos Proto‐Oncogene, AP‐1 Transcription Factor Subunit
FPR2: Formyl Peptide Receptor 2
GADD45B: Growth Arrest and DNA Damage Inducible Beta
GEO: Gene Expression Omnibus
GO: Gene Ontology
GZMA: Granzyme A
HBB: Haemoglobin Subunit Beta
HLA‐B: Major Histocompatibility Complex, Class I, B
HLA‐DPB1: Major Histocompatibility Complex, Class II, DP Beta 1
HLA‐DQB1: Major Histocompatibility Complex, Class II, DQ Beta 1
HLA‐DRA: Major Histocompatibility Complex, Class II, DR Alpha
HLA‐DRB1: Major Histocompatibility Complex, Class II, DR Beta 1
HSP90AA1: Heat Shock Protein 90 Alpha Family Class A Member 1
HSPA1A: Heat Shock Protein Family A (Hsp70) Member 1A
HSPA1B: Heat Shock Protein Family A (Hsp70) Member 1B
HSPA6: Heat Shock Protein Family A (Hsp70) Member 6
HSPA6: Heat Shock Protein Family A (Hsp70) Member 6
HSPB1: Heat Shock Protein Family B (Small) Member 1
HSPD1: Heat Shock Protein Family D (Hsp60) Member 1
HSPE1: Heat Shock Protein Family E Member 1
HSPH1: Heat Shock Protein Family H (Hsp110) Member 1
I/R: Ischemia/Reperfusion
ICAM1: Intercellular Adhesion Molecule 1
IER3: Immediate Early Response 3
IL1B: Interleukin 1 Beta
Imm‐DEGs: Immune‐related Differentially Expressed Genes
IRF1: Interferon Regulatory Factor 1
IRI: Ischemia/Reperfusion Injury
JUN: Jun Proto‐Oncogene, AP‐1 Transcription Factor Subunit
JUND: Jun D Proto‐Oncogene
KCs: Kupffer Cells
KEGG: Kyoto Encyclopedia of Genes and Genomes
LITAF: Lipopolysaccharide Induced TNF Factor
LSECs: Liver Sinusoidal Endothelial Cells
LSECs: Liver Sinusoidal Endothelial Cells
LT: Liver Transplantation
Ly6C: Lymphocyte Antigen 6 Complex, Locus C
MALAT1: Metastasis Associated Lung Adenocarcinoma Transcript 1
MCP‐1/CCL2: Monocyte Chemoattractant Protein‐1 / Chemokine (C‐C motif) Ligand 2
MIF: Macrophage Migration Inhibitory Factor
MMP19: Matrix Metalloproteinase 19
MRC1: Mannose Receptor C‐Type 1
MT1M: Metallothionein 1 M
MTHFD2: Methylenetetrahydrofolate Dehydrogenase (NADP+ Dependent) 2
NAMPT: Nicotinamide Phosphoribosyltransferase
NDUFB4: NADH:Ubiquinone Oxidoreductase Subunit B4
NFKBIZ: Nuclear Factor Kappa B Subu
NK/T: Natural Killer/T Cells
NR4A2: Nuclear Receptor Subfamily 4 Group A Member 2
NUCB1: Nucleobindin 1
PBMCs: Peripheral Blood Mononuclear Cells
PP: Pre‐procurement
PPI: Protein–Protein Interaction
PR: Post‐Reperfusion
PTPRC: Protein Tyrosine Phosphatase, Receptor Type C
RNA‐seq: RNA Sequencing
RPS20: Ribosomal Protein S20
RPS29: Ribosomal Protein S29
SAT1: Spermidine/Spermine N1‐Acetyltransferase 1
scRNA‐seq: Single‐cell RNA Sequencing
SIK1: Salt Inducible Kinase 1
SOCS3: Suppressor Of Cytokine Signalling 3
SOCS3: Suppressor of Cytokine Signalling 3
SOD2: Superoxide Dismutase 2
SRGN: Serglycin
THBS1: Thrombospondin 1
TM4SF1: Transmembrane 4 L Six Family Member 1
TNFRSF12A: Tumour Necrosis Factor Receptor Superfamily Member 12A
TSC22D3: TSC22 Domain Family Member 3
VCAN: Versican
VIM: Vimentin
VPS28: Vacuolar Protein Sorting 28 Homologue
ZNF331: Zinc Finger Protein 331
ZNF429: Zinc Finger Protein 429

## 1 Introduction

In multicellular organisms, cellular communication frequently relies on a myriad of molecules, encompassing cell factors and membrane proteins [1, 2]. This intricate network of molecules collaborates harmoniously to govern biological activities and uphold the organism's optimal functionality. Notably, receptor–ligand‐mediated intercellular communication has emerged as a pivotal mechanism orchestrating diverse biological processes, including but not limited to development, differentiation and disease progression [3, 4]. By mediating the interaction between cells, receptor–ligand dynamics empower the exchange of vital information that is essential for sustaining precise cellular function and preserving tissue homeostasis [5, 6, 7].

Hepatic ischemia/reperfusion injury (IRI) is a common occurrence during liver surgeries, such as liver transplantation (LT), resection and trauma [8]. IRI directly jeopardises liver viability, posing a substantial challenge to achieving favourable outcomes [9, 10]. The ischemia/reperfusion (I/R) process induces substantial liver damage and initiates an inflammatory response through intricate signalling pathways that involve diverse cell types, including hepatocytes, sinusoidal endothelial cells, Kupffer cells (KCs), neutrophils, macrophages and platelets [11, 12, 13]. However, there is currently a critical knowledge gap regarding the intricate cellular interactions and molecular mechanisms driving hepatic IRI owing to the heterogeneity of immune cells and non‐immune cells in the liver [14]. These pathological events mutually reinforce each other, resulting in severe or even irreversible liver dysfunction. Thus, it is crucial to develop strategies that break this detrimental cycle and effectively mitigate IRI. Recent strides in single‐cell RNA‐sequencing (scRNA‐seq) technologies have transformed our capacity to comprehensively explore organ ecosystems and fathom the cellular heterogeneity inherent in diseases. Researchers have unveiled intrinsic crosstalk among immune subsets across various diseases by applying scRNA‐seq to scrutinise immune cell populations [15, 16, 17]. Although some studies have used scRNA‐seq to delve into the context of hepatic IRI [18], our grasp of the all‐encompassing landscape of cell crosstalk in this particular injury remains limited. Further, it is imperative to attain a more extensive understanding of the cellular interactions and molecular processes driving hepatic IRI.

In summary, an in‐depth comprehension of the cellular and molecular dimensions by collecting liver tissue samples pre‐procurement (PP), at the end of preservation (EP) and 2 h post‐reperfusion (PR) can reveal the intricate interactions among distinct cell types and identify the pivotal molecular entities engaged in this progression. In this atlas, the annotated cell subgroups and interactions between different cell subpopulations during LT will play a pivotal role in advancing innovative therapeutic approaches aimed at mitigating liver injury and elevating patient prognosis.

## 2 Methods

### 2.1 Data Processing for scRNA‐Seq

The scRNA‐seq dataset of GSE171539 was sourced from the Gene Expression Omnibus (GEO) database, yielding a single‐cell transcriptomic atlas comprising 14,313 liver cells from a 47‐year‐old donor and a 51‐year‐old recipient. This dataset encompasses single‐cell gene expression data obtained from liver grafts both before and after LT. All of the samples underwent RNA extraction and analysis utilising 10× Genomics and Illumina sequencing technology. Subsequent data processing steps were conducted using R software (version 3.6.1) and the Seurat package (version 3.1.2) [19].

### 2.2 Identification of Differentially Expressed Genes (DEGs) in Specific Cell Types

We used CellPhoneDB, an accessible database of receptor–ligand interactions, in order to explore the interplay between distinct cell subtypes in hepatic IRI [20]. Mean values and *p*‐value calculations were derived using CellPhoneDB. The correlation strength between specific cell types was assessed based on the overall mean and the number of interactions.

### 2.3 Analysis of Cell Crosstalk

To explore the interplay between distinct cell subtypes in hepatic IRI, we employed CellPhoneDB [20], an accessible database of receptor–ligand interactions. Mean values and *p*‐value calculations were derived utilising CellPhoneDB. The correlation strength between specific cell types was assessed based on the overall mean and the number of interactions.

### 2.4 Hepatic IRI‐Related Microarray Data Source and Pre‐Processing

We obtained four distinct datasets from the GEO database (GSE14951, GSE12720, GSE23649 and GSE7706). We de‐batched the four abovementioned datasets using the R package sva in order to construct the integrated GEO dataset [21].

### 2.5 Analysis of DEGs From Four Gene Expression Profiles

We used the ‘limma’ package in R [22] to conduct a differential gene analysis on the integrated dataset, aiming to identify genes exhibiting varying expression between diseased and control samples. The primary focus of this study was to explore the effect of immune‐related gene expression levels on hepatic IRI. Differential genes were identified based on thresholds of |log_2_FC| > 1.5 and adj. *p* < 0.05, signifying upregulated DEGs. Conversely, genes with log_2_FC < −1.5 and adj. *p* < 0.05 were categorised as downregulated DEGs. The outcomes of differential gene expression analysis were visually represented through volcano plots and heatmaps. Identification of differentially expressed immune genes was achieved via intersecting DEGs and immune genes.

### 2.6 Determining Molecular Subtypes by Analysing Crucial Immunomodulators

For the identification and validation of clusters, we used a resampling‐based method known as consistency clustering. This method was applied to ascertain the membership and subgroup numbers within each cluster. To explore diverse immunological patterns utilising significant immune‐related differentially expressed genes (Imm‐DEGs), we used the ConsensusClusterPlus package in R [23].

### 2.7 Construction of Regulatory Network

The STRING database (http://string‐db.org) is a widely used resource for identifying known proteins and predicting their interactions. In this study, we used the STRING database to construct a protein–protein interaction (PPI) network connecting DEGs, Imm‐DEGs and potential immune genes. The PPI network model was visualised using Cytoscape (v3.7.2) [24], and functional annotations of the genes within the network were conducted using closeGO [25].

### 2.8 Functional Enrichment Analysis Using HALLMARK, GO and KEGG Analyses

The HALLMARK pathway analysis method is employed for the exploration of gene expression data to uncover shared biological functions or pathways within gene sets. The process typically involved several steps: Conducting enrichment analysis on the gene expression matrix, comparing the expression of genes within each gene set across samples to discern which pathways or functions are significantly enriched under the study conditions. Results interpretation: Interpreting the outcomes of the enrichment analysis to identify pathways or functions significantly regulated under the study conditions and elucidating their relevance to the research topic.

To enhance our comprehension of DEGs, we conducted Gene Ontology (GO) and Kyoto Encyclopedia of Genes and Genomes (KEGG) pathway analyses. GO annotation was performed using the clusterProfiler R package, and KEGG pathway analysis was done using the ggplot2 package [20]. These analyses assisted in revealing the biological processes and pathways associated with the identified DEGs.

### 2.9 Immune Cell Infiltration Profile

In our research, we used the CIBERSORT algorithm to assess the presence of 22 distinct immune cell types within each sample [26]. To ensure accuracy in evaluating immune infiltration, a significance level of *p* < 0.05 was set and applied for initial sample analysis. This threshold was maintained throughout subsequent data analysis processes. Subsequently, using a Wilcoxon test, we compared the proportions of immune cells across various groups. This method enabled us to discern noteworthy disparities in immune cell compositions among the studied groups.

### 2.10 Consensus Clustering

To conduct an unsupervised clustering analysis of immune‐related patients, considering DEGs, we applied the ‘ConsensusClusterPlus’ algorithm [23]. This algorithm used various metrics, such as the cumulative distribution function curve, consensus score and consensus matrix. These metrics collectively aid in determining the optimal number of subtypes, denoted as ‘*k*’.

### 2.11 Immunofluorescence of CD31, CD14, ANXA1 and FPR2 in Five LT Tissues Following IRI

In this study, we investigated the variations in CD31, CD14, ANXA1 and FPR2 expression in liver transplant donors before and after transplantation using immunofluorescence techniques. Liver tissue samples were obtained and thinly sliced for preparation. Following tissue blocking to prevent nonspecific binding, primary antibodies targeting CD31 (ab9498, 1:500; Abcam), CD14 (AP6294A, 1:25; abcepta), ANAX1 (BM3925, 1:100; Boster) and FPR2 (A8351, 1:500; Abcam) were applied and allowed to bind overnight. Subsequently, unbound antibodies were washed away, and fluorescent secondary antibodies were applied to detect the bound primary antibodies. Optional counterstaining with a nuclear stain was performed for visualising cell nuclei. The tissue sections were then mounted onto slides for microscopy. Fluorescence imaging was conducted to analyse changes in expression levels of CD31, CD14, ANXA1 and FPR2 following IRI.

### 2.12 Mouse Ischemia–Reperfusion Injury Model

We utilised 7‐ to 8‐week‐old male C57BL/6 mice to establish the ischemia–reperfusion injury (IRI) model. Following anaesthesia, a midline abdominal incision exposed the liver, which was then mobilised and its associated ligaments and mesentery were transected. Vascular clamps were applied to blockage the blood supply to the left lateral and median lobes for 45 min before releasing to allow reperfusion. Mice were sacrificed 12 h PR to evaluate hepatic damage.

### 2.13 Western Blot Analysis

Protein extraction from tissue samples was conducted using RIPA buffer, and protein concentrations were determined by the BCA assay. SDS‐PAGE (10%) was employed to resolve proteins, which were then transferred to a PVDF membrane for immunoblotting with primary antibodies at 4°C overnight, followed by incubation with secondary antibodies for 2 h. Chemiluminescence was used for detection. The primary antibodies used included FPR2, β‐tubulin and a goat anti‐rabbit IgG secondary antibody.

### 2.14 Flow Cytometry

C57BL/6J mice were euthanized with carbon dioxide, and a prechilled HBSS buffer containing digestive enzymes and DNase was used for cell preparation. Cells were adjusted to a density of 5–10 × 10^6^ cells/mL for flow cytometry analysis. After resuspension in staining buffer, cells were labelled with antibodies against CD45 (ab210225, Abcam), CD11b (ab133357, Abcam) and Ly6G (ab238132, Abcam) to identify monocytes. Forward and side scatter gating, along with CD45, CD11b and Ly6G expression, were used to quantify the monocyte population within the liver tissue.

### 2.15 Statistical Analysis

For normally distributed data, a two‐tailed Student *t*‐test compared differences between two groups to determine if their means were significantly different. Results were presented as means ± standard deviations (SDs) to show central tendency and variability. Significance levels were denoted as *p < 0.05 and **p < 0.01, indicating statistically significant differences between groups at these respective levels. GraphPad Prism 8.0 and SPSS 21.0 were used for statistical analysis.

## 3 Results

### 3.1 Landscape of the Cell Composition in Hepatic IRI

**FIGURE 1:**
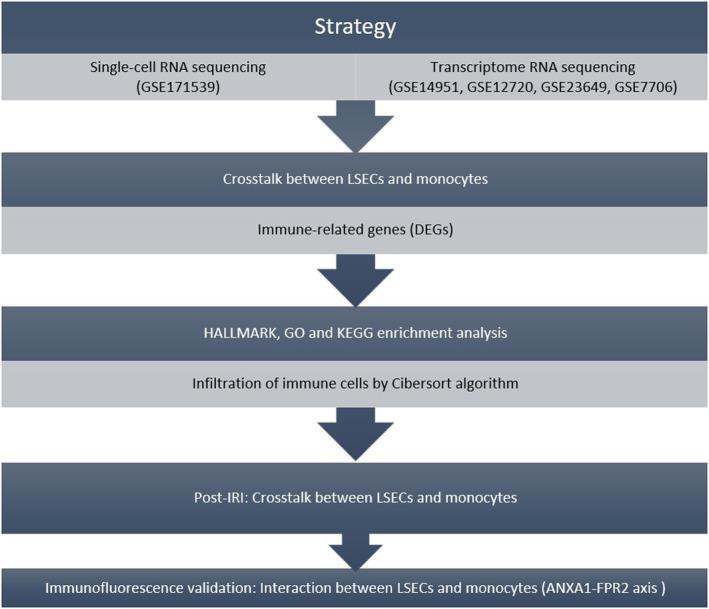
Workflow. The workflow aimed to comprehensively investigate the intricate crosstalk between LSECs and monocytes in hepatic IRI following LT.

**FIGURE 2:**
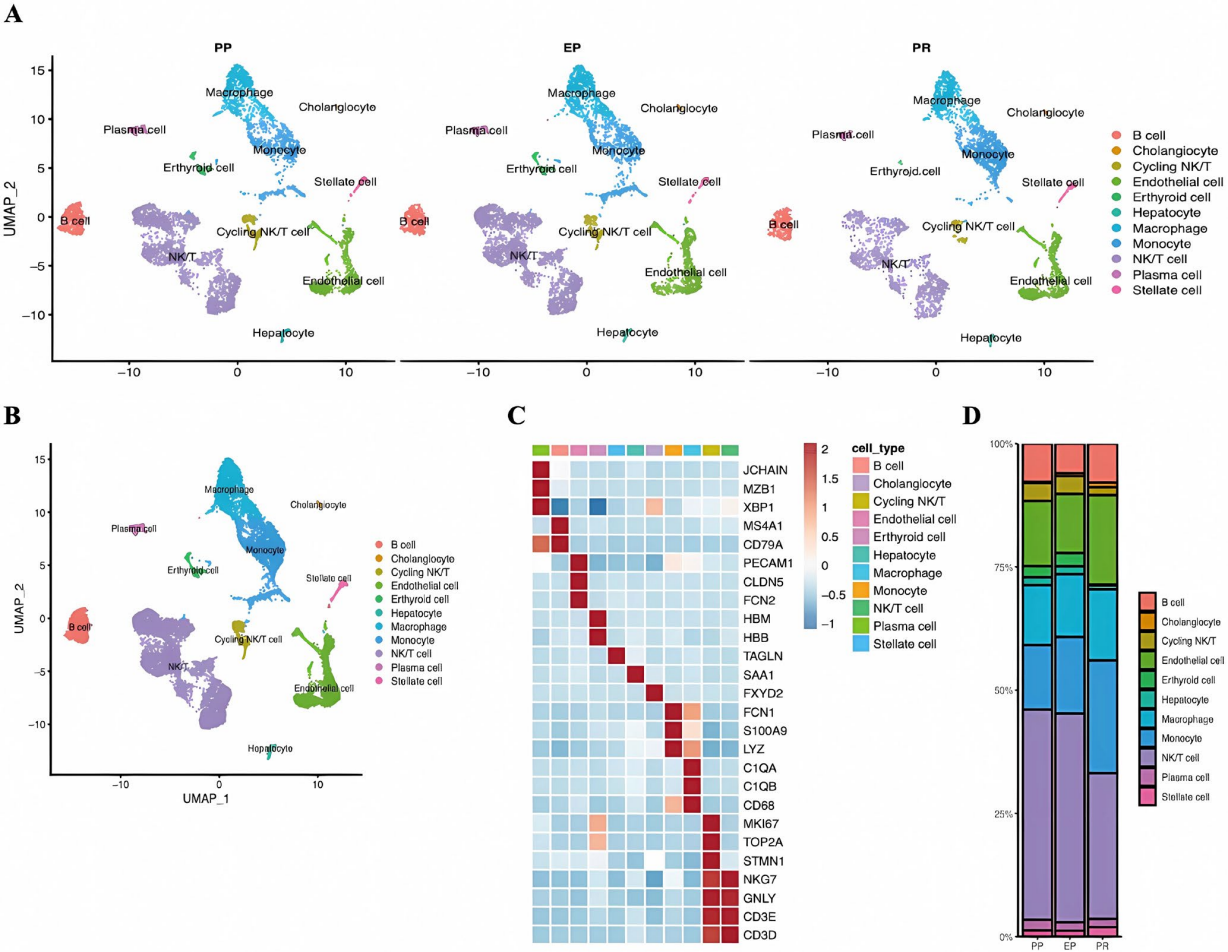
Landscape of the cell composition in hepatic IRI. (A) Overview of the cell clusters based on scRNA‐seq data from three time points in hepatic IRI tissues (UMAP). (B) Frequency of cells in each cluster. (C) Heatmap showing the expression of marker genes in the indicated cell types. (D) Histogram indicating the proportion of cells. Comparative analysis of cell type proportions at three time points—PP, EP and 2 h PR—showed an increased proportion of LSECs and monocytes in PR tissues compared to PP tissues.

The bioinformatics analysis in this study followed the process outlined in Figure 1. The scRNA‐Seq dataset from GSE171539 was obtained from the GEO database. After undergoing initial quality control filtering, the scRNA‐seq data included a total of 14,313 cells. These cells were then clustered into 25 groups using UMAP, and singleR was used to categorise them into distinct cell types. Consequently, 11 cell types were identified within these 25 clusters, comprising five non‐immune cell types and six immune cell types (Figure 2A,B). Non‐immune cell populations were primarily composed of endothelial cells (PECAM1, CLDN5 and FCN2), hepatic stellate cells (TAGLN), erythroid cells (HBM and HBB), cholangiocytes (FXYD2) and hepatocytes (SAA1) (Figure 2C). In contrast, the immune cell population was predominantly characterised by macrophages (FCN1, S100A9, LYZ, C1QA, C1QB and CD68), NK/T cells (NKG7, GNLY, CD3E and CD3D), cycling NK/T cells (MKI67, TOP2A, STMN1, NKG7, GNLY, CD3E and CD3D), monocytes (FCN1, S100A9 and LYZ), B cells (MS4A1 and CD79A) and plasma cells (JCHAIN, MZB1, XBP1 and CD79A) (Figure 2C).

Further, a comparison was conducted regarding the proportions of each cell type at three different time points: PP, EP and 2 h PR. The findings revealed increased proportion of LSECs and monocytes in PR tissues compared with PP tissues (Figure 2D). In summary, we explored the cellular composition landscape using a methodology similar to that of a prior study [18].

### 3.2 DEGs in Specific Cell in Hepatic IRI From scRNA‐Seq Analysis

**FIGURE 3:**
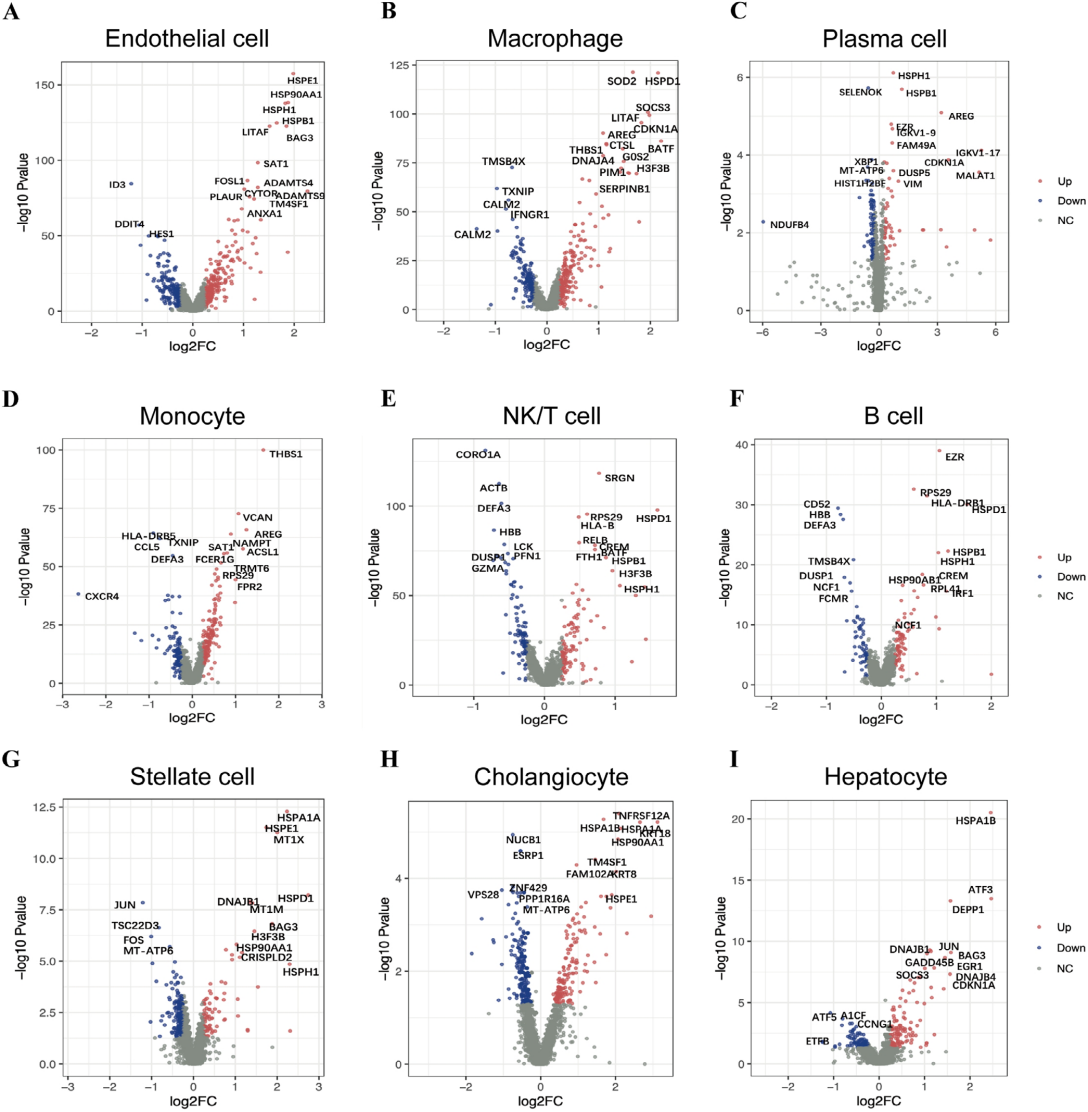
DEGs in specific cell in hepatic IRI from scRNA‐seq analysis. Differentially expressed genes (DEGs) in immune cells and non‐immune cells in the liver (A–I). Red dots represent upregulated genes, and blue dots represent downregulated genes. Summarised DEGs in various liver cell types identified through single‐cell sequencing, including endothelial cell, macrophage, monocyte, plasma cells, monocytes, NK/T cells, B cells, stellate cells, cholangiocytes and hepatocytes, highlighting genes implicated in inflammatory responses and cellular homeostasis.

We next identified the DEGs in non‐immune cells and immune cells between PP and PR tissues, focusing on the most significantly dysregulated genes. Subsequently, our attention turned to immune cells that exhibited notable changes in tumour tissues, particularly LSECs and monocytes. Our analysis of LSECs revealed 14 upregulated genes and three downregulated genes in PR tissues (Figure 3A). Notably, HSPE1, HSP90AA1, HSPH1 and HSPB1 have previously been associated with the heat shock protein signalling pathway. In addition, BAG3, SAT1 and CYTOR were identified, with prior studies linking these genes to disruptions in the cell cycle and disturbances in the homeostasis of LSECs. Other DEGs, including LITAF, TM4SF1 and ANXA1, implicated in inflammatory responses, may influence endothelial cell characteristics and contribute to LT injury.

In terms of macrophages, we observed a set of 20 DEGs, with 15 upregulated genes and 5 downregulated genes (Figure 3D). These DEGs were involved in intricate signalling pathways specific to macrophages. Notably, FPR2, LITAF and BATF played crucial roles in pathways related to immune regulation. SOD2 was associated with antioxidant stress response pathways, HSPD1 was linked to heat shock protein‐related pathways, and CDKN1A was involved in regulating the cell cycle, among others. These genes played significant roles in mediating the biological processes in which monocytes were engaged.

In addition, we summarised the genes with aberrant expression identified through single‐cell sequencing in other types of liver cells as follows: plasma cell (HSPH1, HSPB1, AREG, FAM49A, DUSP5, NDUFB4, VIM and MALAT1) (Figure 3C); monocyte (THBS1, VCAN, AREG, NAMPT, ACSL1, SAT1, CCL5 and CXCR4) (Figure 3D); NK/T cell (SRGN, RPS29, HSPD1, HLA‐B, HSPB1, HSPH1, CARO1A, DEFA3, GZMA and DUSP1) (Figure 3E); B cell (EZR, RPS20, HLA‐DRB1, HSPD1, HSPB1, CD52, HBB and DEFA3) (Figure 3F); stellate cell (HSPA1A, HSPE1, MT1M, BAG3, HSP90AA1, JUN, TSC22D3 and FOS) (Figure 3G); cholangiocyte (TNFRSF12A, HSPA1B, TM4SF1, HSPE1, NUCB1, ESRP1, VPS28 and ZNF429); and hepatocyte (HSPA1B, ATF3, JUN, DEPP1, BAG3, DNAJB1, GADD45B, SOCS3, CDKN1A, ATF5 and A1CF) (Figure 3H,I).

### 3.3 Cell–Cell Communication in Hepatic IRI

**FIGURE 4:**
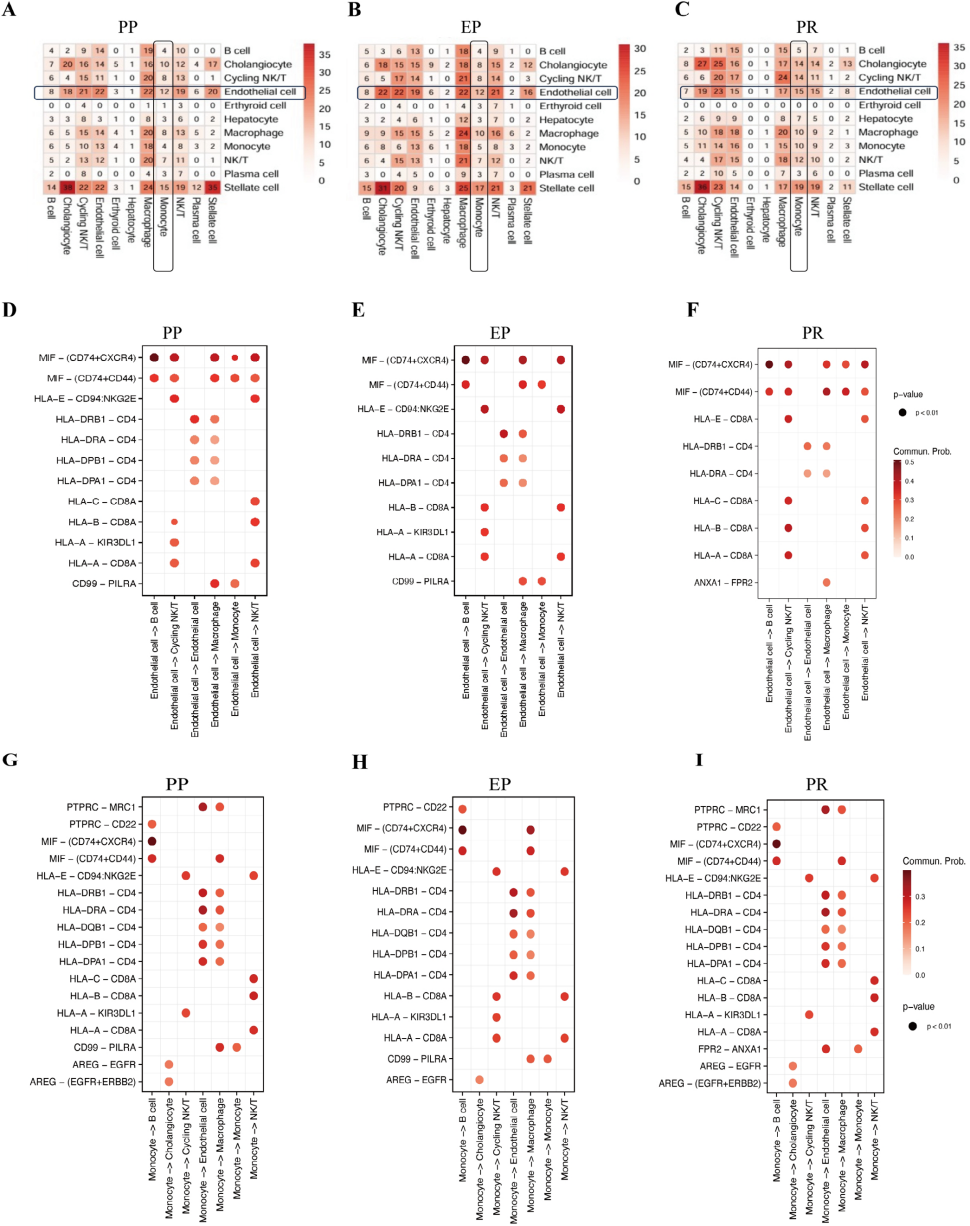
Cell crosstalk in hepatic IRI. (A–C) We conducted a comparative analysis of scRNA‐seq data obtained at three distinct time points (PP, EP and PR) during LT, specifically examining alterations in intercellular communication at the PR time point. Ans the bidirectional communication between LSECs and monocytes unveiled a strengthened communication pathway involving ANXA1‐FPR2. The strength of cell crosstalk was measured as the total mean and number of interactions. Results revealed heightened communication between monocytes and liver cells, particularly emphasising enhanced interactions with endothelial cells and hepatic stellate cells during the PR stage. (D–I) Cell crosstalk based on ligand–receptor interaction in LSECs and monocytes (significant mean > 0.1).

To investigate the intercellular communication in PP and PR tissues, we used CellPhoneDB to analyse receptor–ligand interactions. This analysis focused on examining the correlation between cell A (x‐axis) and cell B (y‐axis) based on their mean and number of interactions. In the initial phase of our investigation, we noted a heightened presence of monocytes and LSECs within liver tissues both pre‐ and post‐LT. Moreover, our research revealed that with the escalating quantities of monocytes and LSECs, the communication and interplay between these cell populations were significantly bolstered. These findings indicate that this phenomenon may underscore a pivotal mechanism in liver IRI (Figure 4A–C).

From the perspective of monocytes, we identified heightened signalling pathways facilitating communication with other liver cells, including PTPRC‐MRC1, PTPRC‐CD22, MIF‐(CD74 + CXCR4), HLA‐DRB1‐CD4, HLA‐DRA‐CD4, HLA‐DQB1‐CD4, HLA‐DPB1‐CD4, AREG‐EGFR and more. Our investigation into the bidirectional communication between LSECs and monocytes unveiled a strengthened communication pathway involving ANXA1‐FPR2. This enhancement suggests that as LSECs are activated, the augmented ANXA1‐FPR2 receptor communication may further amplify the chemotactic, infiltrative and activating properties of monocytes (Figure 4D–I).

### 3.4 Immune‐Related DEGs in Hepatic IRI From Transcriptome RNA Sequencing Datasets

In addition to uncovering cellular and genetic changes before and after LT through scRNA analysis, we made the decision to delve deeper into the modifications in inflammatory responses at the cellular and genetic levels before and after LT using multiple RNA‐sequencing perspectives. This approach represented a pioneering effort in conducting a comprehensive analysis of IRI associated with LT through a multi‐omics framework.

**FIGURE 5:**
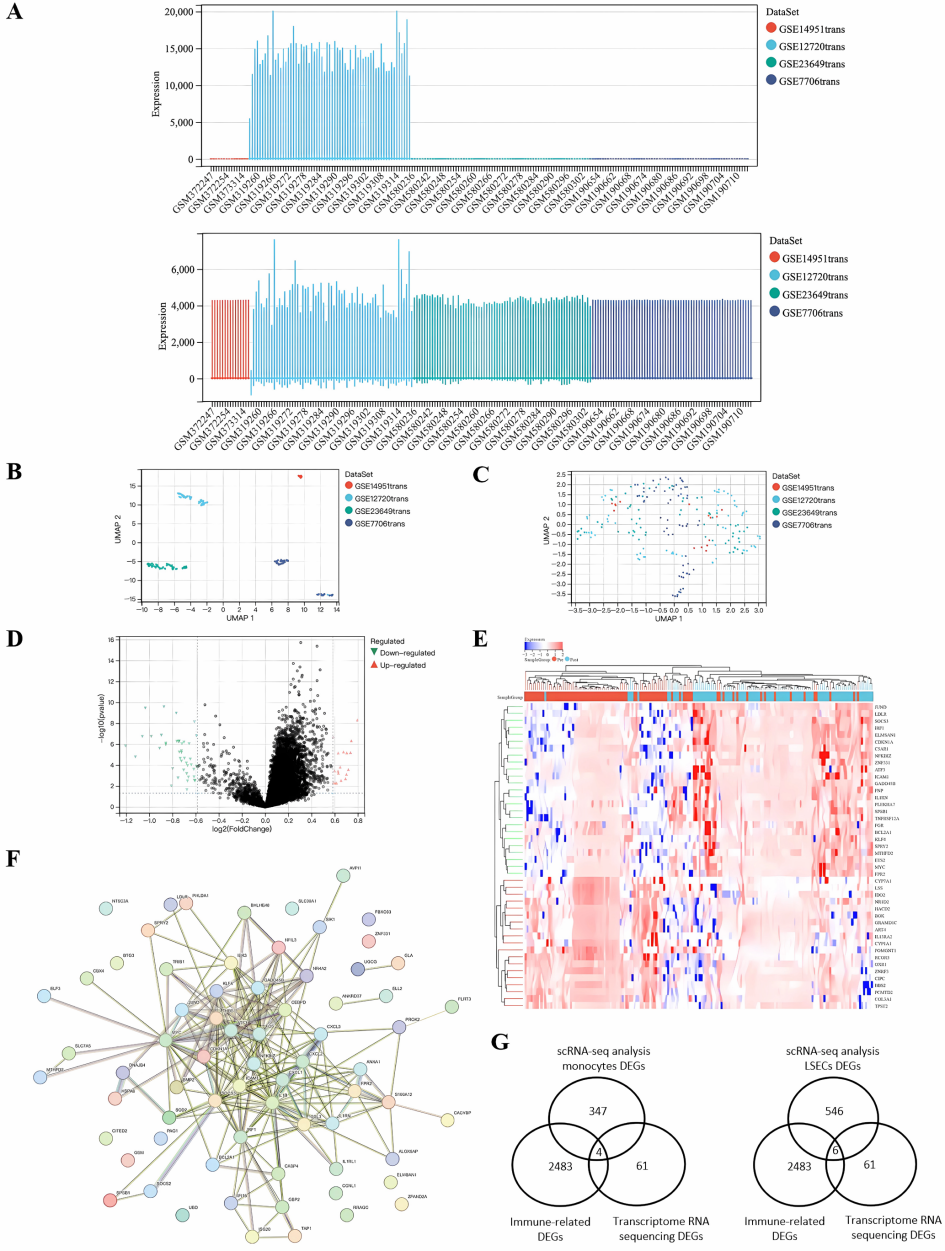
Immune‐related DEGs in hepatic IRI. De‐batching of GEO data was performed. (A) Statistics of gene expression levels in the dataset before and after de‐batching. (B, C) PCA of the integrated datasets before and after de‐batching. Integrated dataset creation for the comparison of gene expression between 80 transplanted and 18 donor livers, accounting for batch effects from transcriptome RNA sequencing datasets sourced from the GEO database. (D) Volcano plot of hepatic IRI‐related differentially expressed genes (DEGs). (E) Heatmap of IRI‐related DEG expression levels: Pink indicated pre‐reperfusion samples, green indicates post‐reperfusion samples, red indicated high gene expression, and blue indicates low gene expression. Volcano and heatmap plots depicting the differential expression analysis, which identified 61 immune‐related DEGs, with 19 upregulated and 42 downregulated genes before and after liver transplantation (LT). (F) DEG PPI network: Protein–protein interaction (PPI) networks constructed from the DEGs, highlighting the central roles of ANXA1 and FPR2 as hub genes within these networks. Yellow nodes indicate Imm‐DEGs. (G) Immune gene Venn diagram: Identification of immune‐related genes associated with monocytes (SOCS3, JUND and FPR2, NR4A2) and LSECs (ICAM1, SOCS3, NFKBIZ, JUND, TNFRSF12A and HSPA6) through integration of single‐cell sequencing analysis data with 2483 immune‐related genes from the ImmPort database.

We created an integrated dataset (Figure 5A–C) and compared gene expression in 80 transplanted livers to that in 18 donor livers when removing batch effects of transcriptome RNA sequencing datasets from the GEO database. From these four RNA‐seq profiles, we conducted an analysis of the differential expression of immune‐related genes before and after LT. The differential analysis identified 61 DEGs, consisting of 19 upregulated and 42 downregulated genes volcano and heatmap (Figure 5D,E). Some genes (FPR2, NFKBIZ and ICAM1) exhibited higher expression levels after LT (Figure 5E). Furthermore, we constructed PPI networks using the DEGs (Figure 5F).

Through single‐cell sequencing analysis, we identified differential gene expression in monocytes and LSECs before and after LT, encompassing 347 and 546 genes, respectively. Subsequent RNA‐sequencing analysis revealed 61 genes exhibiting differential expression. Integration of this data with 2483 immune‐related genes from the ImmPort database led to the identification of 4 immune‐related genes associated with monocytes (SOCS3, JUND, FPR2 and NR4A2) and 6 immune‐related genes linked to LSECs (ICAM1, SOCS3, NFKBIZ, JUND, TNFRSF12A and HSPA6) (Figure 5G).

Notably, we verified the interaction index between ANXA1 and FPR2 through network queries, with an annotation score of 0.99 for their interaction. Interestingly, ANXA1 and FPR2 emerged as the hub genes in their respective PPI networks.

### 3.5 Identification of Immune‐Related Subgroup Based on DEGs

**FIGURE 6:**
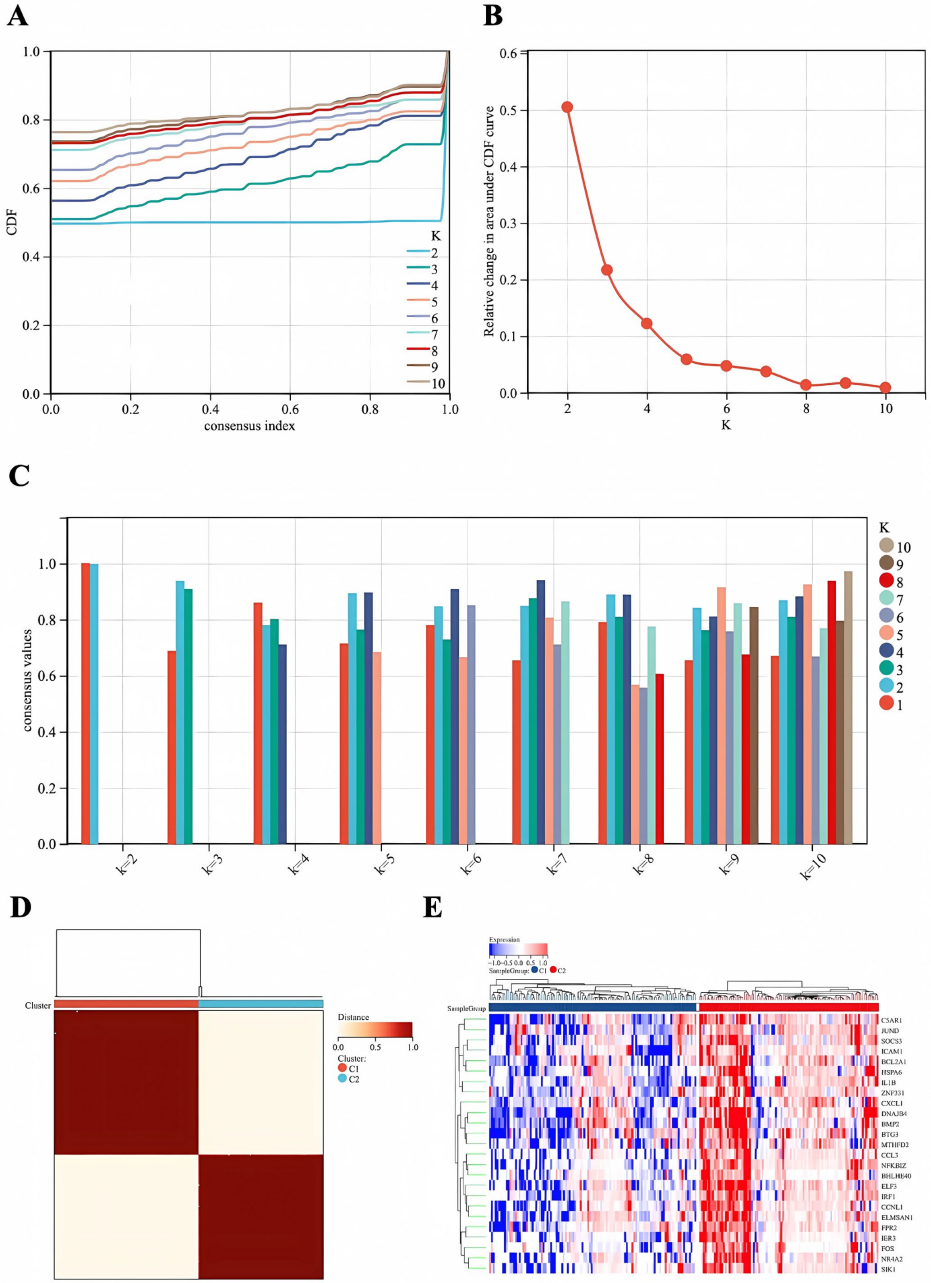
Identification of immune‐related subgroup based on DEGs. (A) Representative cumulative distribution function (CDF) curve. (B) Representative CDF delta area curve. (C) Consensus clustering score when *k* is 2–9. (D) Utilising the ConsensusClusterPlus package in R, we identified two immunological subgroups, C1 and C2, characterised by distinct differential gene expression patterns following ischemia–reperfusion injury (IRI). Cluster C1 showed a decrease in gene expression, while C2 exhibited an increase. The optimal *k* value of 2 was confirmed by the maximum consensus score. (E) Principal component analysis (PCA) confirmed the efficacy of 25 DEGs in differentiating between the two subgroups. Heatmaps illustrated the expression patterns of immune genes, with genes such as C5AR1, JUND, SOCS3, ICAM1 and others being significantly upregulated in the C2 subgroup compared to C1.

Using the ConsensusClusterPlus package in R, we identified two distinct immunological patterns known as cluster C1 and cluster C2. These clusters represent different levels of differential gene expression, with C1 subgroup showing a noticeable decrease in gene expression after IRI and C2 subgroup exhibiting a significant increase. The optimal grouping, achieved by setting *k* = 2, was supported by the consensus score reaching its maximum value (Figure 6A–D). In addition, principal component analysis (PCA) demonstrated that these 25 DEGs effectively differentiated between the two subgroups (Figure 6E). Subsequently, heatmaps were generated to depict the distinct expression patterns of immune genes in the two groups (Figure 6A–D). The expression levels of C5AR1, JUND, SOCS3, ICAM1, BCL2A1, HSPA6, IL1B, ZNF331, CXCL1, DNAJB4, BMP2, BTG3, MTHFD2, CCL3, NFKBIZ, BHLHE40, ELF3, IRF1, CCNL1, ELMSAN1, FPR2, IER3, FOS, NR4A2 and SIK1 were significantly higher in C2 subgroup than in cluster C1 subgroup (Figure 6E).

### 3.6 HALLMARK, GO and KEGG Pathway Analyses

**FIGURE 7:**
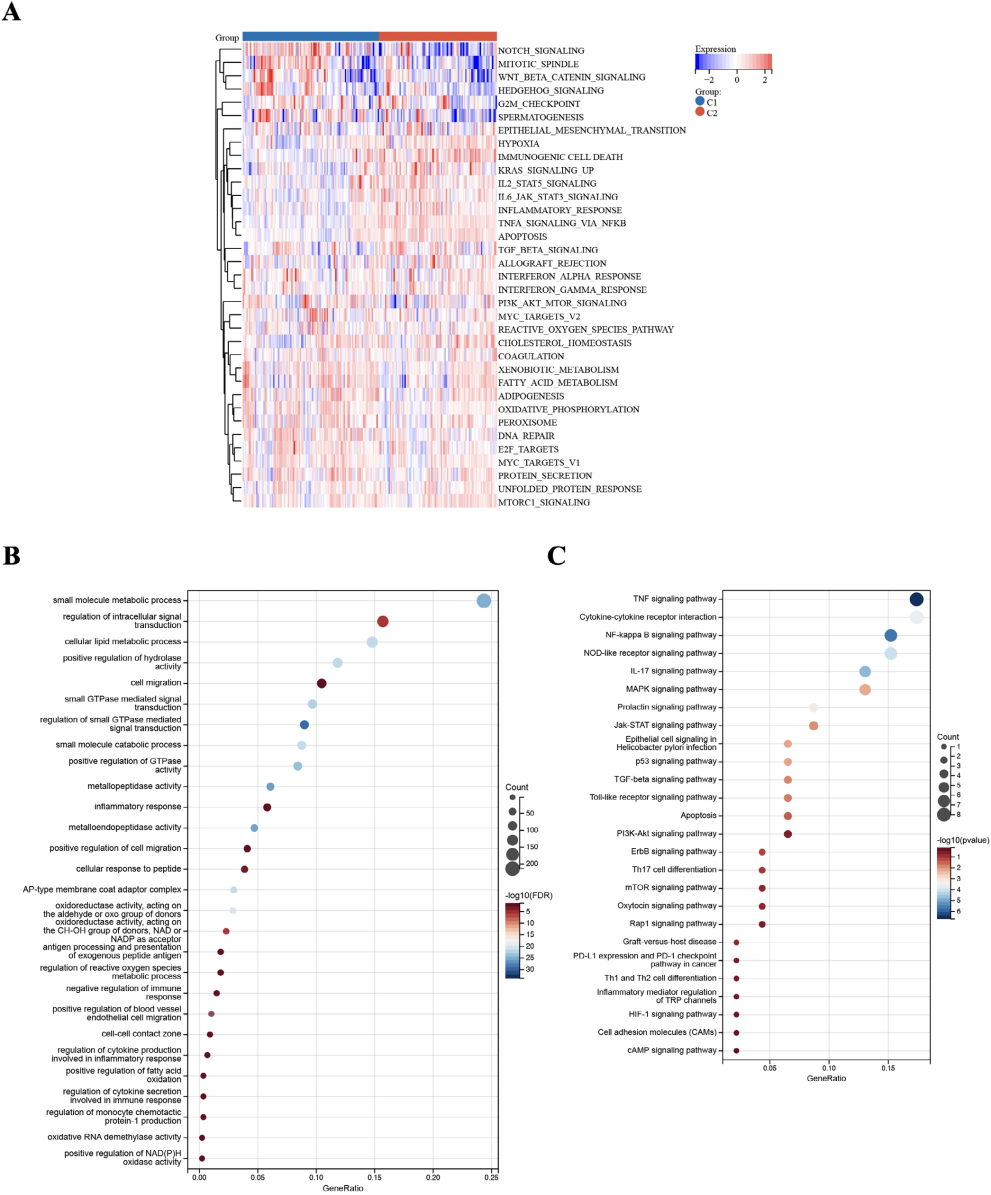
Gene set enrichment analysis. (A) Heatmap showing the correlation between the enrichment score of differentially enriched HALLMARK pathway. The weakly correlated group showed negative regulation in pathways such as Notch signalling, mitotic spindle, Wnt‐β‐catenin signalling, G2/M checkpoint and spermatogenesis. (B, C) Gene set enrichment analysis (GSEA) between two different immune models. GO and KEGG pathway enrichment analyses in post‐ischemia (PR) tissues revealed significant enrichment in immune cell functions related to intracellular signal transduction, cell migration, inflammatory response, cellular response to peptides, antigen processing and presentation and regulation of cytokine production in the inflammatory response.

To better comprehend the molecular mechanisms of highly expressed immune‐related genes, we performed an in‐depth functional enrichment analysis using HALLMARK pathway analysis. Within the strongly correlated immune group, we observed an elevation in signalling pathways associated with hypoxia, immunogenic cell death, KRAS signalling up, IL2‐STAT5 signalling, TNFA signalling via NF‐KB and inflammatory responses. Conversely, the weakly correlated group displayed negative regulation in pathways such as Notch signalling, mitotic spindle, Wnt‐β‐catenin signalling, G2/M checkpoint and spermatogenesis (Figure 7A).

To gain deeper insight into the biological alterations occurring in PR tissues, we conducted GO and KEGG pathway enrichment analyses (Figure 7B,C). Our analysis revealed that immune cells exhibited significant enrichment in functions related to the regulation of intracellular signal transduction, cell migration, inflammatory response, cellular response to peptides, antigen processing and presentation as well as the regulation of cytokine production involved in the inflammatory response.

### 3.7 Variations of Immune Characteristics in C1/C2 Subgroup Signatured at Differential Immune‐Related DEGs

**FIGURE 8:**
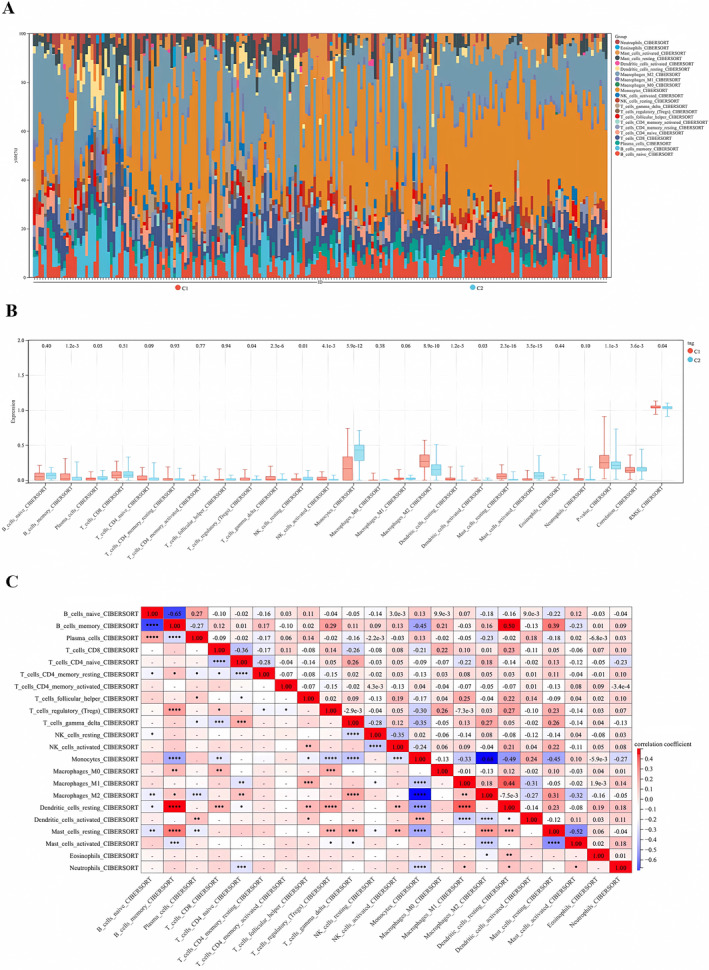
Variations in immune characteristics among C1 and C2 subgroups. (A) Box plots were used to illustrate the differences in the proportions of 22 infiltrating immune cells between the C1 and C2 subgroups. The CIBERSORT algorithm revealed a significant increase in monocytes within the inflammation‐associated C2 subgroup compared to the inflammation‐inhibiting C1 subgroup. (B) An immune cell content histogram showed the distribution of 22 immune cells along the horizontal axis and their content along the vertical axis; the C1 subgroup was indicated in pink and the C2 subgroup in blue. Notably, the C2 subgroup demonstrated a marked increase in monocytes (***p < 0.001) and a distinct decrease in the expression of M2‐type macrophages (***p < 0.001), suggesting a reduction in immune‐tolerant M2 macrophages and a potential exacerbation of the inflammatory response following IRI. (C) Another immune cell content histogram displayed the positive correlation between the increase in monocyte population and activated dendritic cells, indicating a close relationship between monocyte infiltration and antigen presentation in the immune response following IRI. Conversely, a notable negative correlation between monocytes and M2 macrophages was observed, suggesting their potential role in the differentiation of subsequent M1 macrophages and further contributing to the inflammatory response following IRI. *p < 0.05, **p < 0.01, ***p < 0.001, ****p < 0.0001.

By employing the CIBERSORT algorithm, our analysis revealed a pronounced increase in monocytes within the inflammatory‐related C2 subgroup when compared to the inflammatory‐inhibiting C1 subgroup (Figure 8A). Further scrutiny of the differential expression ratios of these cells uncovered a significant disparity. Notably, within the C2 subgroup, there was a marked increase in monocytes (*p* < 0.001). Furthermore, a distinct decrease in the expression of M2‐type macrophages was noted within the immune‐inflammatory‐related C2 subgroup (*p* < 0.001). This observation hints at a reduction in immune‐tolerant M2 macrophages, thereby exacerbating the inflammatory response post‐IRI (Figure 8B).

Our findings indicated that as the monocyte population increased, there was a positive correlation with activated dendritic cells. It suggested a close relationship between monocyte infiltration and expansion with antigen presentation in the immune response post‐IRI. While a notable negative correlation existed between monocytes and M2 macrophages, indicating potential involvement in the differentiation of subsequent M1 macrophages and further contributing to the inflammatory response post‐IRI (Figure 8C).

### 3.8 Expression Variations of ANXA1 and FPR2 in Single‐Cell and Bulk RNA Sequencing

**FIGURE 9:**
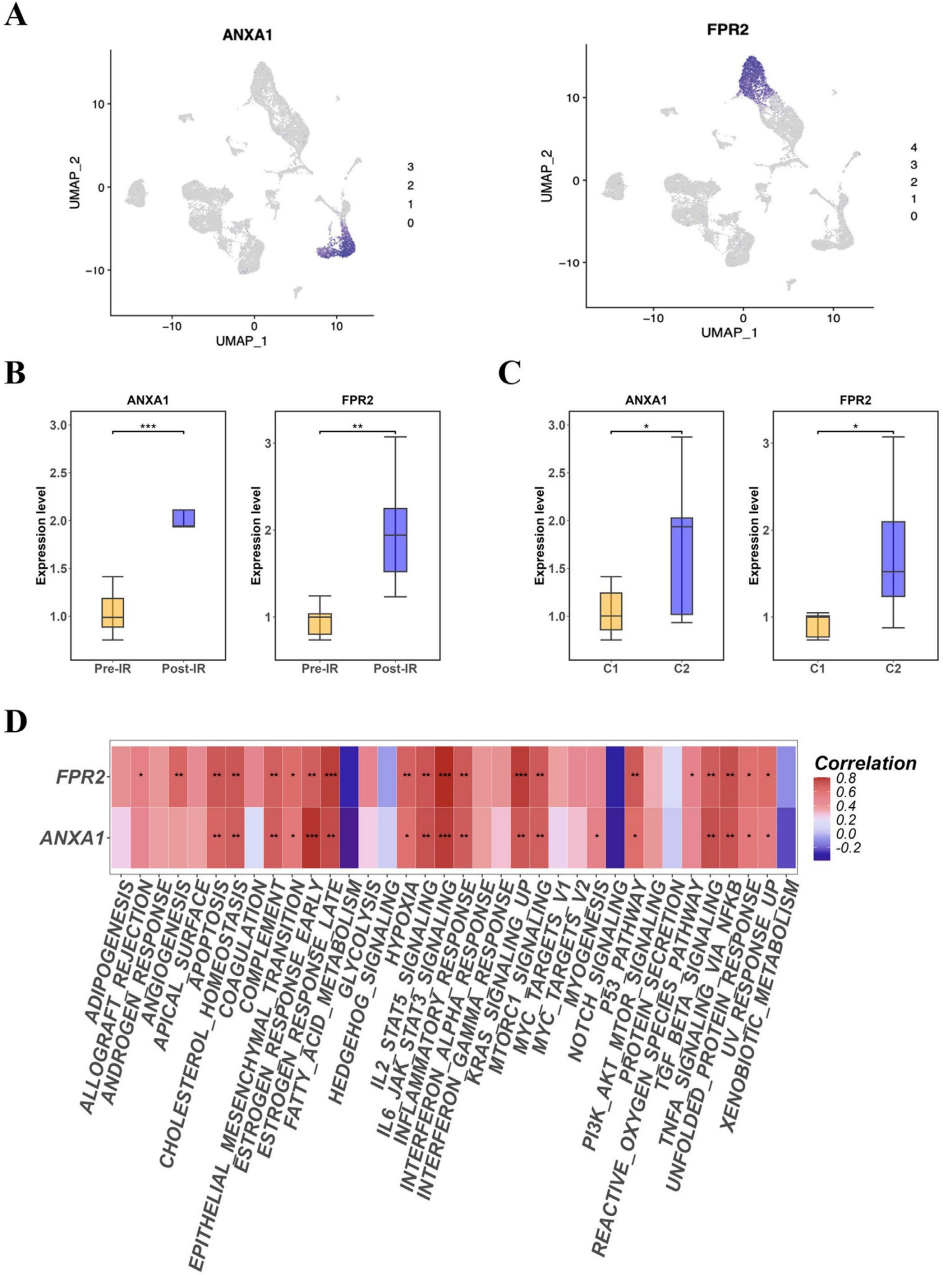
Analyse the expression of ANXA1 and FPR2 in both single‐cell data and bulk RNA datasets. (A) A UMAP expression plot for ANXA1/FPR2 at the single‐cell level. Single‐cell RNA sequencing data revealed upregulation of ANXA1 and FPR2 in liver sinusoidal endothelial cells and monocytes following ischemia–reperfusion injury; (B) The differential expression of ANXA1/FPR2 between pre‐IR and post‐IR subgroups at the bulk‐RNA level. Bulk RNA sequencing data showed significant overexpression of ANXA1 and FPR2 in the Post‐IR group compared to the Pre‐IR group, with *p*‐values < 0.05; (C) The differential expression of ANXA1/FPR2 between subtypes C1 and C2. Stratification into C1 and C2 subgroups based on immune‐inflammatory responses revealed increased expression of ANXA1 and FPR2 in the C2 subgroup (indicative of inflammatory processes) relative to the C1 subgroup, with statistical significance (*p* < 0.05); (D) A heatmap of the correlation between ANXA1/FPR2 expression and GSVA pathway scores. Pearson correlation analysis using the GSVA package in R4.3.2 demonstrated significant correlations between ANXA1 and FPR2 expression levels and various pathway scores. *p < 0.05, **p < 0.01, ***p < 0.001, ****p < 0.0001.

In our investigation, we initially focused on the scRNA sequencing data to discern the upregulation of ANXA1 and FPR2 following IRI, which was specifically associated with liver sinusoidal endothelial cells and monocytes (Figure 9A). The bulk RNA sequencing data indicated that when comparing the expression levels of ANXA1 and FPR2 between the Pre‐IR and Post‐IR groups, both genes were significantly overexpressed in the Post‐IR group, as determined by *t*‐tests with *p*‐values < 0.05, showing consistency between the two datasets (Figure 9B). Further analysis was conducted by stratifying the data into C1 and C2 subgroups based on distinct immune‐inflammatory responses. We concentrated on the C2 subgroup, which is indicative of inflammatory processes and observed an increased expression of ANXA1 and FPR2 relative to the C1 subgroup, with statistical significance (*p* < 0.05) (Figure 9C). Utilising the GSVA package in R4.3.2 for HALLMARK gene set enrichment analysis, we performed a Pearson correlation analysis to evaluate the relationship between the expression levels of ANXA1 and FPR2 and various pathway scores. This analysis revealed significant correlations between these genes and multiple pathway scores, underscoring their potential roles in pivotal biological processes (Figure 9D).

### 3.9 Determination of the Expression of ANXA1 and FPR2 in LT Patients

**FIGURE 10:**
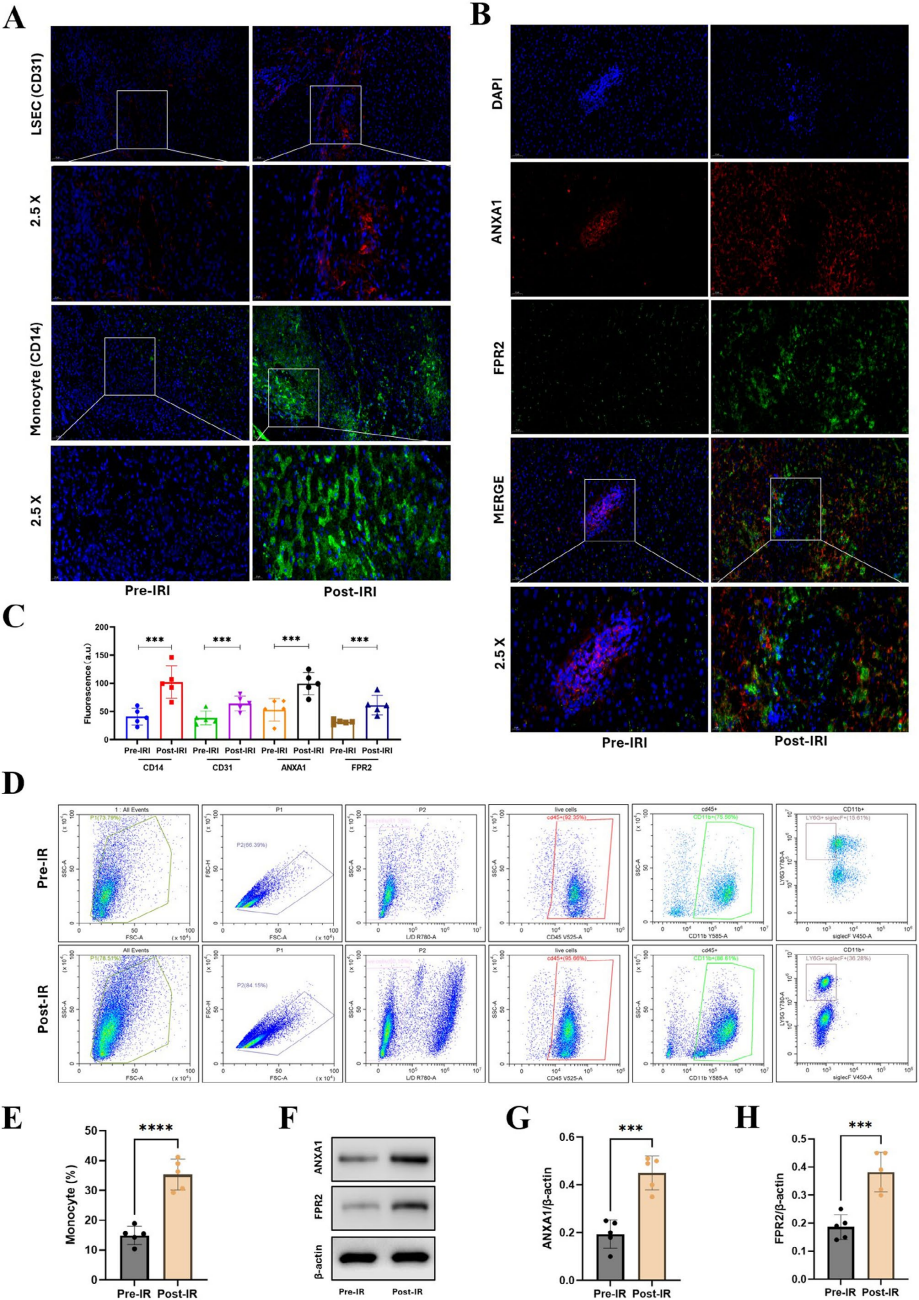
Validation of ANXA1 and FPR2 in LT patients tissue with immunofluorescence. (A) Immunofluorescence showed increased expression of LSECs (CD31) and monocytes (CD14) following liver transplantation, supported by staining and scRNA‐seq data. (B, C) Immunofluorescence revealed a significant increase in the expression of CD31, CD14, ANXA1 and FPR2 following ischemia–reperfusion (post‐IR) (*p* < 0.001). (D, E) Flow cytometry compared the changes in monocytes within a mouse liver IR model, finding a marked elevation in monocytes post‐IR (*p* < 0.01). (F–H) Western blot further examined the protein expression of ANXA1 and FPR2 in the mouse liver IR model, showing that both proteins had significantly higher expression post‐IR compared to pre‐IR (*p* < 0.01). *p < 0.05, **p < 0.01, ***p < 0.001, ****p < 0.0001.

We conducted immunofluorescence staining experiments on tissue samples from five liver transplant patients. Initially, we compared the expression changes of monocytes (CD14 marker) and LSECs (CD31 marker) before and after LT. Our findings revealed a substantial increase in red fluorescence around the hepatic sinusoids marked by CD31 (*p* < 0.001) and a noticeable enhancement in green fluorescence around the hepatic sinusoids marked by CD14 (*p* < 0.001) 2 h after LT. This indicates an augmented expression of monocytes and hepatic sinusoidal endothelial cells following the graft unveiling, which is consistent with our single‐cell sequencing results (Figure 10A).

Furthermore, we examined the expression of ANXA1 and FPR2. ANXA1 exhibited increased expression following the activation of LSECs, while FPR2, a receptor expressed on the surface of monocytes, indicated their activation and involvement in regulating inflammatory signalling pathways. Our experimental results demonstrated a significant enhancement in the expression of both ANXA1 and FPR2 (both *p* < 0.001), aligning with our scRNA‐seq findings (Figure 10B,C).

To further delve into the changes in monocytes within the mouse liver IR model, we employed flow cytometry to quantify the alterations in monocyte populations. We observed that the proportion of monocytes was 15.6% before the injury, and this percentage rose to 36.28% post‐IR, indicating a significant increase (*p* < 0.01) (Figure 10D,E). Concurrently, we utilised western blot analysis to evaluate the protein levels of ANXA1 and FPR2, and the outcomes confirmed a notable elevation in the protein expression of both ANXA1 and FPR2 following the liver IR injury (*p* < 0.01) (Figure 10G,H). These findings underscore the dynamic responses of monocytes and the upregulation of ANXA1 and FPR2 in the context of liver IR injury.

## 4 Discussion

Monocytes and macrophages are instrumental in managing inflammatory reactions within a diverse range of pathological conditions. Our study, along with recent research, has highlighted the complex roles that monocytes and macrophages play within the immune system [27]. In inflammatory bowel disease (IBD), monocyte chemoattractant protein‐1 (MCP‐1/CCL2) orchestrated the migration of peripheral monocytes towards the inflamed intestinal mucosa by interacting with the CCR2 receptor expressed on monocytes. The recruitment of monocytes set off a cascade of pro‐inflammatory cytokines, ultimately resulting in uncontrolled mucosal inflammation and damage [28]. In the lungs, interstitial macrophages (IM) underwent continual replenishment as peripheral blood monocytes from the bone marrow migrate into the lungs. These monocytes further differentiated to maintain the stability of the IM population [29]. During the fibrotic progression of non‐alcoholic fatty liver disease/non‐alcoholic steatohepatitis (NAFLD/NASH), monocytes differentiated into macrophages that influenced liver fibrosis, providing a basis for developing targeted therapies [30]. Furthermore, studies have indicated that transcriptional repressors ETV3 and ETV6 played a pivotal role in promoting the differentiation of monocytes into dendritic cells (mo‐DCs) by inhibiting macrophage differentiation [31]. Additionally, during the progression of acute liver failure, a marked increase in the subset characteristics and proportion of liver monocytes/macrophages was observed, leading to macrophage re‐programming, a key pathogenic mechanism underlying the severe systemic inflammatory responses characteristic of acute liver failure [32].

**FIGURE 11:**
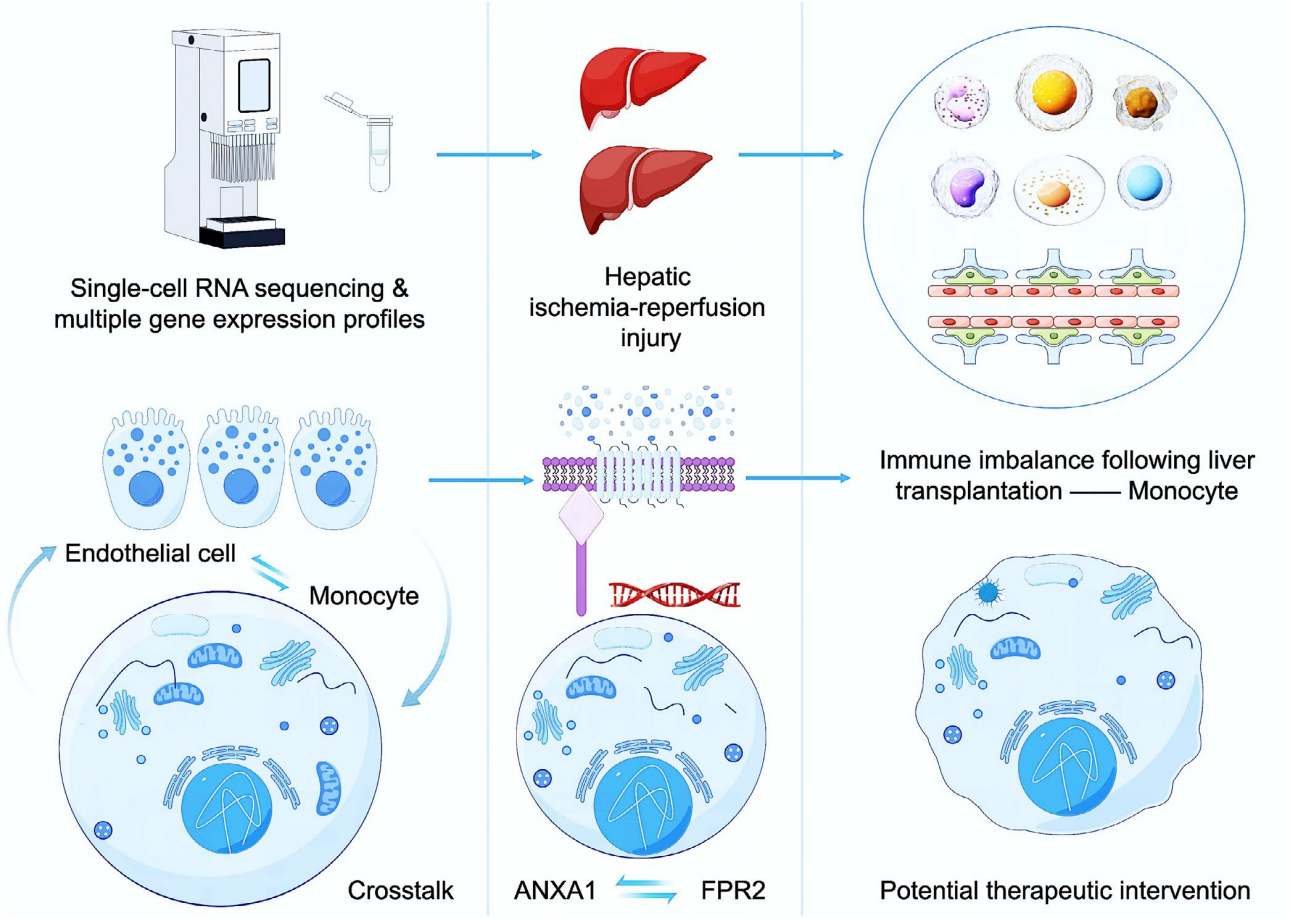
Summary. The intricate crosstalk between LSECs and monocytes has the potential to initiate a cascade of events, activating monocytes and fueling inflammatory responses, thereby exacerbating the detrimental effects of hepatic IRI.

How monocytes contribute to the modulation of disease progression and inflammation is an area that necessitates more profound exploration. The interaction between monocytes and tissue cells is believed to be pivotal in the inflammatory process [33]. Recent researches have focused on understanding interactions between monocytes and resident parenchymal cells within tissues [34, 35, 36, 37]. For example, during pulmonary fibrosis progression, there was a notable increase in MMP19 expression in alveolar epithelial cells. MMP19 interaction with ET1 promoted epithelial‐to‐mesenchymal transition (E(nd)MT) and facilitated monocyte infiltration into lung tissue, worsening bleomycin (BLM)‐induced pulmonary fibrosis [38]. Insights from scRNA‐seq highlighted the ongoing interplay between CD63+ LSECs and Ly6C+ monocytes within the hepatic microenvironment of mice. The interaction was crucial in the transition from acetaminophen (APAP)‐induced liver injury (AILI) to acute liver failure [39]. Monocytes played a crucial role in maintaining immune homeostasis through cytokine secretion and interaction with other cells, impacting the development of autoimmune and inflammatory diseases. Currently, research is exploring their therapeutic potential for immune‐based treatments targeting various immune‐related disorders (Figure 11).

In LT, hepatic IRI possessed a significant challenge due to the heightened inflammatory response. By leveraging single‐cell sequencing alongside four bulk RNA‐sequencing datasets, we uncovered significant differential gene expression and cellular interactions that were altered prior to and following liver IRI. This integration of single‐cell and bulk RNA data revealed a strengthened interaction between LSECs and monocytes/macrophages, as depicted in Figure 4. Moreover, in our differential gene expression analysis, both datasets—scRNA and bulk RNA—demonstrated an upregulation of ANXA1/FPR2 expression. This was evident on the single‐cell level in the UMAP (Figure 9A) and also observed in the context of IR injury and inflammation‐related subgroups (subtypes C1 and C2) at the bulk‐RNA level (Figure 9B,C). Collectively, these observations indicated that the expression of ANXA1 and FPR2 was markedly increased, serving as a key differential marker post‐IR injury. To substantiate our findings, we constructed a mouse model of IR injury and utilised flow cytometry to assess the changes in monocyte populations before and after IR. We observed a notable increase in the proportion of monocytes post‐procedure, rising from 15.6% to 36.2% (*p* < 0.01). Immunofluorescence further confirmed the enhanced expression of CD31 (marking LSECs) and CD14 (marking monocytes) following the procedure. Additionally, western blot analysis was employed to evaluate the expression levels of ANXA1/FPR2 in our mouse model of IR injury, revealing a significant increase in protein expression for both (*p* < 0.01), aligning with the findings from our single‐cell and bulk RNA data. In conclusion, our high‐throughput data analysis revealed that the enhanced interaction between monocytes and liver sinusoidal endothelial cells, along with the upregulated expression of ANXA1/FPR2, are among the distinguishing features before and after liver IR injury. By constructing a mouse model of IR injury, we further validated the expression changes of monocytes and ANXA1/FPR2, with results that were consistent with our high‐throughput data (Figure 11).

Monocytes were massively recruited to the liver following IRI, and these cells could further exacerbate the inflammatory response by releasing a variety of inflammatory mediators, such as cytokines and chemokines [40]. LSECs were also damaged during the IRI process, which could lead to their dysfunction, thereby affecting liver blood circulation and immune regulation [18]. The role of monocytes in IRI was double‐edged. On one hand, they could clear damaged cells and debris, promoting repair; on the other hand, excessive inflammatory responses could lead to further tissue damage [41]. LSECs were not only directly injured in IRI but might also further recruit and activate monocytes by releasing inflammatory mediators and extracellular matrix components, forming a positive feedback loop [42]. In LT, IRI was an inevitable process, and its severity directly affected patient outcomes. Therefore, understanding the specific roles of monocytes and LSECs in IRI could aid in the development of new interventions to reduce the occurrence and severity of IRI [43]. For instance, inhibiting monocyte recruitment or enhancing the protective function of LSECs might help alleviate the inflammatory response following IRI, improving the function of the transplanted liver and patient survival rates [44].

ANXA1 and its receptor FPR2 played significant roles in a variety of inflammatory diseases. Studies demonstrated that ANXA1 mediated anti‐inflammatory and pro‐resolving effects by binding to FPR2, thereby alleviating IRI [45]. Specifically, ANXA1 could protect tissues from injury by inhibiting the activation and migration of neutrophils and reducing the release of inflammatory mediators. Although the role of the ANXA1‐FPR2 signalling pathway in IRI was partially understood, IRI in the context of LT was a complex pathological process involving multiple cellular and molecular mechanisms. Within our gene enrichment analysis as depicted in Figure 7, we observed the enhancement of several signalling pathways within the C2 subgroup, which is associated with immunological differentially expressed genes (imm‐DEGs). Notably, pathways including the Toll‐like receptor signalling pathway, cell migration and regulation of reactive oxygen species were found to be significantly upregulated. Other potential pathways or mechanisms included: The TLR4 signalling pathway, where TLR4 (Toll‐like receptor 4) played a key role in IRI, particularly in DCD (donation after cardiac death) LT. The activation of TLR4 could trigger a robust inflammatory response, leading to tissue damage. Research found that the TLR4 inhibitor TAK242 significantly improved IRI after DCD LT [46]. HMGB1‐mediated NET formation, where HMGB1 (high mobility group box 1) was a crucial inflammatory mediator that could promote the formation of neutrophil extracellular traps (NETs), further exacerbating IRI. Studies showed that the interaction between HMGB1 and NETs in acute rejection (AR) might be one of the important mechanisms of IRI [47]. The Notch1 signalling pathway, which played a vital role in modulating immune cell functions and inflammatory responses. Research indicated that Notch1 in macrophages could control hepatocyte necroptosis mediated by TAK1 and RIPK3, thus affecting the development of IRI [48]. Further clarification was needed in the future regarding the specific mechanisms of the ANXA1‐FPR2 signalling pathway in different cell types, such as KCs and endothelial cells.

FPR2 could potentially exert dual regulatory effects on immune modulation, with its impact varying across different immune microenvironments. ANXA1, secreted by LSECs in microvesicles, interacted with FPR2 on neutrophils, thereby enhancing neutrophil activation within liver tissue [49]. Additionally, Liu et al. proposed that the activation of FPR2 suppressed M2 macrophage polarisation, consequently fostering tumour growth in oesophageal cancer [50]. Interestingly, Ansari's research on sickle cell disease revealed that ANXA1‐induced activation of macrophage FPR2/ALX axis, leading to differentiation of M2 polarisation and conferring a protective effect [51]. In our investigation, we observed a significant upregulation of FPR2 following hepatic IRI. By integrating the findings from scRNA‐seq, it was postulated that FPR2 may play a pivotal role in regulating monocyte‐related immune responses. The discovery suggested that FPR2 could possessed a multifaceted and critical function in shaping the immune response subsequent to LT, necessitating further research efforts to elucidate its precise regulatory mechanisms of hepatic IRI. Nevertheless, there is a need for more in‐depth experimental validation regarding the subsequent research on FPR2, particularly in understanding how it modulates monocyte participation in inflammatory responses and the mechanisms involved. This aspect represents a gap in our current investigation.

## 5 Conclusion

Our research substantially enhanced our comprehension of the fundamental mechanisms associated with LT IRI, providing valuable insights for future investigations. Further exploration of these insights may unveil new targets for therapeutic intervention, ultimately enhancing outcomes for patients undergoing LT.

## Author Contributions

**Chao Sun:** conceptualization (equal), investigation (equal), methodology (equal), writing – original draft (equal). **Li Li:** investigation (equal), methodology (equal), resources (equal), software (equal), writing – original draft (equal). **Dan Li:** investigation (equal), supervision (equal), validation (equal), writing – review and editing (equal). **Zhengxin Wang:** data curation (equal), funding acquisition (equal), supervision (equal), writing – review and editing (equal).

## Conflicts of Interest

The authors declare no conflicts of interest.

## Data Availability

The data supporting the findings of this study are available within the article. All of the raw data used in this study can be obtained from the corresponding author upon reasonable request.
